# Four Decades of Military Posttraumatic Stress: Protocol for a Meta-analysis and Systematic Review of Treatment Approaches and Efficacy

**DOI:** 10.2196/33151

**Published:** 2021-10-25

**Authors:** Jenny J W Liu, Anthony Nazarov, Bethany Easterbrook, Rachel A Plouffe, Tri Le, Callista Forchuk, Alec Brandwood, Kate St Cyr, Edouard Auger, Ken Balderson, Mathieu Bilodeau, Amer M Burhan, Murray W Enns, Patrick Smith, Fardous Hosseiny, Gabrielle Dupuis, Maya Roth, Natalie Mota, Vicky Lavoie, J Don Richardson

**Affiliations:** 1 The MacDonald Franklin Operational Stress Injury Research Centre Lawson Health Research Institute London, ON Canada; 2 Department of Psychiatry Schulich School of Medicine & Dentistry Western University London, ON Canada; 3 Department of Psychiatry and Behavioural Neurosciences McMaster University Hamilton, ON Canada; 4 Dalla Lana School of Public Health University of Toronto Toronto, ON Canada; 5 Clinique pour traumatismes liés au stress opérationnel Centre intégré universitaire de santé et de services sociaux de la Capitale-Nationale Quebec, QC Canada; 6 Department of Psychiatry and Neurosciences Faculty of Medicine Laval University Quebec, QC Canada; 7 St. Joseph's Operational Stress Injury Clinic Toronto, ON Canada; 8 Ontario Shores Centre of Mental Health Sciences Whitby, ON Canada; 9 Department of Psychiatry Faculty of Medicine University of Toronto Toronto, ON Canada; 10 Department of Psychiatry Rady Faculty of Health Sciences University of Manitoba Winnipeg, MB Canada; 11 Winnipeg Operational Stress Injury Clinic Winnipeg, ON Canada; 12 Centre of Excellence on Post-Traumatic Stress Disorder and Related Mental Health Conditions Ottawa, ON Canada; 13 Yeates School of Graduate Studies Ryerson University Toronto, ON Canada; 14 Department of Clinical Health Psychology University of Manitoba Winnipeg, ON Canada; 15 St. Joseph's Operational Stress Injury Clinic London, ON Canada

**Keywords:** military personnel, psychotherapy, pharmacotherapy, stress disorders, posttraumatic, meta-analysis, systematic review, therapy, stress, disorder, posttraumatic stress disorder, review, treatment, efficacy, military, Canada, veteran

## Abstract

**Background:**

Over 85% of active members of the Canadian Armed Forces have been exposed to potentially traumatic events linked to the development of posttraumatic stress disorder (PTSD). At the time of transition to civilian life, as high as 1 in 8 veterans may be diagnosed with PTSD. Given the high prevalence of PTSD in military and veteran populations, the provision of effective treatment considering their unique challenges and experiences is critical for mental health support and the well-being of these populations.

**Objective:**

This paper presents the protocol for a meta-analysis and systematic review that will examine the effectiveness of treatment approaches for military-related PTSD.

**Methods:**

This PROSPERO-preregistered meta-analysis is being conducted in accordance with the PRISMA (Preferred Reporting Items for Systematic Reviews and Meta-Analyses) and Cochrane guidelines. A comprehensive search of the literature was conducted using the databases PsycInfo, Medline, Embase, CINAHL, and ProQuest Dissertation & Theses. Effect sizes will be computed based on changes in PTSD symptom scores over time across studies using validated PTSD scales. A multilevel meta-analysis will examine the overall effects, between-study effects, and within-study effects of available evidence for PTSD treatments in military populations. Effect sizes will be compared between pharmacotherapeutic, psychotherapeutic, and alternative/emerging treatment interventions. Finally, meta-regression and subgroup analyses will explore the moderating roles of clinical characteristics (eg, PTSD symptom clusters), treatment approaches (eg, therapeutic orientations in psychotherapy and alternative therapies and classifications of drugs in pharmacotherapy), as well as treatment characteristics (eg, length of intervention) on treatment outcomes.

**Results:**

The literature search was completed on April 14, 2021. After the removal of duplicates, a total of 12,002 studies were screened for inclusion. As of July 2021, title and abstract screening has been completed, with 1469 out of 12,002 (12.23%) studies included for full-text review. Full review is expected to be completed in the summer of 2021, with initial results expected for publication by early winter of 2021.

**Conclusions:**

This meta-analysis will provide information on the current state of evidence on the efficacy and effectiveness of various treatment approaches for military-related PTSD and identify factors that may influence treatment outcomes. The results will inform clinical decision-making for service providers and service users. Finally, the findings will provide insights into future treatment development and practice recommendations to better support the well-being of military and veteran populations.

**Trial Registration:**

PROSPERO CRD42021245754; https://tinyurl.com/y9u57c59

**International Registered Report Identifier (IRRID):**

DERR1-10.2196/33151

## Introduction

### Background

Over 85% of active members of the Canadian Armed Forces have reported exposure to potentially traumatic events [1], and studies estimate that between 7.5% and 12.9% of veterans are diagnosed with posttraumatic stress disorder (PTSD) on return to civilian life [2,3]. Military-related PTSD may differ from PTSD experienced by civilians [4]. The risk factors, etiology, and prognosis of military-related PTSD are associated with military service, deployment stressors, and unique potentially traumatic events. These events include experiences of combat, moral injury, military sexual trauma, and the LGBTQ Purge [2,5,6]. As a result, PTSD treatment for military and veteran populations may differ in effectiveness from that for nonmilitary populations. Studies have shown that outcomes of both pharmacotherapy and psychotherapy for military-related PTSD have a smaller effect size than those for civilian-related PTSD; military members and veterans have reported poorer response to treatments than civilians [7-9]. In a recent review, Coventry et al [10] noted that while trauma-focused therapies were particularly effective in treating PTSD, the effect was less for military- and veteran-related PTSD.

Given the prevalence of PTSD and the uniqueness of the PTSD experience in military populations, the provision of effective treatment and support is of utmost importance. However, ambiguities and heterogeneities in reports of effectiveness are challenging for service providers [11]. Recent reviews highlight the lack of consensus regarding the trajectory of PTSD, the diversity of approaches in the diagnosis and treatment of PTSD, and the inconsistencies in defining response to PTSD treatments as problematic [10,12-14]. In addition, novel empirical evidence has also underscored service users participating in the treatment decision-making process as additional important determinants of treatment outcomes [4]. Provision of timely, appropriate, and effective treatments and support that are aligned across organizations, service providers, and service users is critical to the well-being of military personnel and veterans. Thus, we aim to provide an overview of the effectiveness of existing treatment options for military-related PTSD.

### Treating Military-Related PTSD

Since the classification of PTSD as a mental disorder in 1980, treatments have evolved to encompass a diversity of approaches, targeting a multitude of symptomology, functioning, and pathways. As a result, clinicians and mental health service providers face the difficult challenge of developing a treatment plan for those diagnosed with PTSD. Current evidence-based treatments can be classified into two categories: psychologically based and pharmacologically based treatments.

The majority of the empirically supported psychological treatments for PTSD fall within the cognitive behavioral therapy framework. Examples of these treatments include cognitive processing therapy [15], trauma-focused cognitive behavioral therapy [16], and prolonged exposure [17]. Outside of the cognitive behavioral therapy framework, another empirically supported treatment for PTSD is eye movement desensitization and reprocessing [18]. In the military context, trauma-focused psychotherapies (prolonged exposure, cognitive processing therapy, and eye movement desensitization and reprocessing) are the most recommended approaches to treating PTSD [19]. These therapies focus on trauma-related negative cognitions and challenging situational and cognitive avoidance as well as on processing the meaning of the trauma. Together, prolonged exposure, cognitive processing therapy, and eye movement desensitization and reprocessing have shown to be most effective in ameliorating PTSD symptoms [19,20]. While much of the effectiveness of these treatments has been evaluated in individual therapy format, there is also increasing empirical support for administering these treatments—specifically cognitive processing therapy—in group format [21]. Some recent studies have also found selective interventions to be noninferior to some trauma-focused therapies, including interpersonal psychotherapy [22] and acceptance and commitment therapy [23].

Pharmacological treatment of PTSD involves the use of various psychotropic medications to target the core symptoms of PTSD, including intrusions, avoidance, negative alterations in cognition and mood, and alteration in arousal and reactivity [24]. As of July 2021, typical pharmacotherapies to treat PTSD include selective serotonin reuptake inhibitors, serotonin and norepinephrine reuptake inhibitors, atypical antipsychotics, β-blockers, and sleep medications (eg, α-blockers, nabilone, hypnotics) [24]. Pharmacological treatments can be categorized by medication typologies, including antidepressants (eg, sertraline), antipsychotics (eg, risperidone), anticonvulsants (eg, topiramate), hypnotics (eg, zopiclone), and mood stabilizers (eg, lithium). In addition to classification according to drug typology, pharmacotherapy treatments can also be categorized by mechanisms of action.

Besides psychological and pharmacological treatments, there are a number of alternative and emerging treatments targeting different aspects of PTSD symptomology. These can include clinical treatments such as deep brain stimulation [25], noninvasive brain stimulation via repetitive transcranial magnetic stimulation, transcranial direct current stimulation [26], and neurofeedback [27]. Emerging therapies may also include cognitive-based conjoint therapy for PTSD [28], animal-assisted therapy [29], and yoga or mindfulness-based therapies [30]. In addition, given the high rates of comorbidities in individuals with PTSD, many approaches have incorporated the treatment of comorbidities to create new combination or adjunctive therapies for the treatment of PTSD [31]. These can include medication-enhanced psychotherapies such as methylenedioxymethamphetamine [32] and virtual reality–based treatments [33].

### Determinants of Treatment Approaches and Clinical Outcomes

A number of review studies have summarized the effectiveness of various treatment approaches. A head-to-head review comparing psychological and pharmacological treatments of combat-related PTSD in 25 studies found that pharmacotherapeutic approaches were slightly more efficacious than psychotherapeutic approaches in ameliorating PTSD symptoms [34]. A network meta-analysis of treatments for PTSD and other mental health conditions stemming from complex trauma drew a contrasting conclusion from the results of 116 studies [10]. The findings suggested that pharmacological interventions were less effective than psychological interventions in the treatment of PTSD and associated functions such as sleep [10]. In addition to disparities across review findings, questions arise, including which factors, if any, influence the effectiveness of various treatment approaches, for whom are different interventions most effective, and what contextual factors, if any, can bolster the effectiveness of treatment approaches for this unique population.

Furthermore, additional effort is needed to expand the scope of reviews. Many reviews included evidence exclusively from randomized controlled trials that often used monotherapies or exclusion criteria [35]. However, the clear-cut criteria applied in research share little overlap with the complexities of real-life practices and experiences of diagnosing and treating PTSD in military and veteran populations. Treatment providers contend with complexities of patient characteristics (eg, chronicity and type of trauma), clinical characteristics of PTSD (eg, symptom clusters, prior treatment or use of medications, and comorbidities), and treatment characteristics (eg, length of treatment, type of treatment and augmentation [13], add-on, and adjunctive treatments) when making treatment-related decisions. In addition, treatment planning is often conducted with patient engagement and feedback in mind [36], and may involve many parallel processes with different health and mental health providers.

### Aims and Objectives

Through a multilevel meta-analytic model, this meta-analysis and systematic review will review the state of evidence on existing treatment options for military-related PTSD and their effectiveness via a preregistered meta-analysis and systematic review. The meta-analysis will serve as a comprehensive scan of the literature while discriminating between effective and ineffective approaches based on considerations of clinical characteristics, treatment characteristics, and individual differences. The systematic review will evaluate the quality of the evidence and examine treatment fidelity, study rigor, and certainty of evidence. Protocols for the meta-analysis and systematic review were developed following the PRISMA (Preferred Reporting Items for Systematic Reviews and Meta-Analyses) guidelines and preregistered on PROSPERO to ensure transparency and replicability [37].

## Methods

### Search Strategy

The literature search was conducted using multiple databases (PsycINFO, PubMed/Medline, Embase, CINAHL, and ProQuest Dissertation & Theses) on April 14, 2021, with a date restriction of 1980. The date restriction represents the first issue of the *Diagnostic and Statistical Manual of Mental Disorders* (DSM) *III*, in which PTSD was officially defined as a distinct diagnosis. In addition to these exploratory databases, we also used PTSDpubs, the JBI Database of Systematic Reviews and Implementation Report, and the Cochrane Library; hand searched for relevant articles via bibliographies; and used known author contact to search for additional titles for potential inclusion.

### Eligibility Criteria

The following criteria were considered for inclusion in the study: (1) adults; (2) military personnel or veterans; (3) individuals with a current diagnosis of PTSD—with etiology due to military service (eg, combat-related PTSD)—under DSM-III, DSM-III-R, DSM-IV, DSM-IV-TR, DSM-5, or International Classification of Diseases criteria; (4) those with some form of incorporated treatment (psychotherapy, pharmacotherapy, alternatives); and (5) those in whom PTSD symptom change was measured via validated measures of PTSD severity (eg, PTSD Checklist for DSM-5). Exclusion criteria were (1) reviews and meta-analyses (though used for known author contact and search); (2) studies with nonadult populations (eg, children, nonhuman); (3) case studies with sample sizes of less than 5; (4) studies without a primary or secondary focus on PTSD in military and veteran populations; and (5) studies with no quantitative data (eg, protocols, corrections, commentaries, and qualitative studies).

### Comparison Groups

While the overall effects of treatments for PTSD will be aggregated and analyzed, the current meta-analysis and systematic review will mainly explore heterogeneities in treatment approaches. These approaches can be broadly categorized as psychological treatments, pharmacological treatments, and alternative/emerging treatments. Psychological treatment is defined as any intervention grounded in the treatment of mental health through individual psychotherapy and delivered by registered mental health professionals. Pharmacological treatments are defined as any therapeutic approaches using prescribed medication(s) as the primary method of treatment. Alternative and emerging treatments include any alternatives and emerging treatments falling outside of the psychological and pharmacological treatment approaches (eg, equine therapy, deep brain stimulation, and ketamine-assisted therapy).

### Measures of Outcomes and Effect

This review will assess changes in PTSD as measured from baseline to postintervention (psychotherapy, pharmacotherapy, or alternative/emerging treatment modality) using validated psychometric scales of PTSD. Measurements taken will report continuous values of PTSD symptomatology and can include the Clinician-Administered PTSD Scale for DSM-5 [38]; the PTSD Checklist for Military and Civilians for DSM-IV [39]; the PTSD Checklist for DSM-5 [40]; the Primary Care PTSD Screen for DSM-5 [41]; the Dissociative Subtype of PTSD Scale [42]; the Posttraumatic Diagnostic Scale for DSM-5 [43]; and the PTSD Symptom Scale [44].

Intervention effects will be examined using mean differences captured via continuous data, and aggregate data will be represented as Hedges *g*, calculated by the differences in means divided by the weighted pooled SD [45]. Hedges *g* combines the SDs of experimental and control groups, resulting in single SD estimates of group differences [46]. Effect sizes will be interpreted based on the recommendation made by Ferguson [46]: Hedges *g* of 0.41 for a minimum effect size representing a practically significant effect in social science, 1.15 for a moderate effect, and 2.70 for a strong effect. In addition to changes in PTSD symptomatology, secondary outcomes will include functional changes related to PTSD, such as quality of life, cognition, and sleep quality, as well as symptoms of commonly reported diagnostic comorbidities of PTSD like major depressive disorder and anxiety disorders.

### Study Identification and Selection

Independent raters will be trained to evaluate studies against eligibility criteria. Studies will be included if they contain continuous PTSD evaluation data collected at the pre- and postintervention stages via validated measures. For the pharmacotherapy group, selection will include a baseline assessment of symptom severity for evaluation of treatment effectiveness followed by the administration of a psychotropic medication. For the psychotherapy group, selection will include a baseline evaluation followed by the administration of a psychologically based treatment. Study reviews are conducted on SWIFT-Active Screener (Sciome), a web-based collaborative screening software for systematic reviews [47]. The reviews will be completed by 8 raters (TL, AB, KS, YL, IK, JS, BJ, and EK). Any disagreements will be resolved through group discussion to reach mutual consensus, led by the first author.

### Data Extraction

From each study, the following data will be extracted: sample size; means and SDs of PTSD scores pre- and post- or mean difference and *P* values; means and SDs of secondary outcome scores pre- and post or mean difference and *P* value (if available); pre- and postcorrelations; type of intervention; moderator variables (if available); clinical characteristics; treatment characteristics; and study characteristics.

Missing data will be handled through author contact. A designated member of the research team will email the corresponding authors or research leads for missing data. A follow-up email will be sent after 1 week over a 2-week response window. All data extracted and received will be recorded via Smartsheet (Smartsheet Inc) and exported to R (R Foundation for Statistical Computing) and Comprehensive Meta-Analysis software (Biostat Inc) for data analysis.

### Strategy for Data Synthesis and Meta-analyses

Using Cochrane’s guide as a framework for data synthesis, the proposed meta-analysis will seek a minimum of 15 studies to be included for overall analysis, and a minimum of 4 studies to be included for subgroup analyses. For each study, pre- and postintervention means and SDs, along with sample size, will be used to calculate effect sizes. Pre- and postintervention correlations will be calculated based on known data and entered for analysis. For studies without pre- and postintervention data, differences between means, paired-group *P* values, and directions of effects found will be used as alternative methods to calculate effect sizes. Data will be analyzed using Comprehensive Meta-Analysis software [48] and the *metafor* R package [49].

The main analyses of the meta-analysis will comprise a multilevel meta-analytic approach to examine dependency among effect sizes of studies, including overall effects, between-study effects, and within-study effects. Overall analysis will compare group-aggregated effects of psychotherapy with group-aggregated effects of pharmacotherapy. Subgroup analysis will be used to examine the moderating role of clinical characteristics (eg, presence and absence of comorbid disorders, PTSD symptom clusters, trauma exposure type, and lifetime diagnosis), treatment characteristics (eg, treatment approach, length, fidelity, and study rigor), and study characteristics (eg, participant demographics, PTSD measurements used, and operationalizations of PTSD). Finally, publication bias will be explored via the visual inspection of funnel plots, the 3-level Egger regression test, the trim-and-fill method, and Orwin fail-safe N.

### Systematic Review and Risk-of-Bias (Quality) Assessment

The systematic review portion of the proposed review will examine intervention fidelity and study rigor. Methods of quality assessment will take into consideration both the quality of studies reported as well as the fidelity of intervention design and delivery. Assessment of fidelity will follow existing frameworks and include benchmarks of design, delivery, receipt, and enactment [50]. Assessment of study rigor will include benchmarks of research design, participant selection, and appropriateness of statistical analysis. Finally, Cochrane’s guide to GRADE (Grading of Recommendations, Assessment, Development and Evaluations) assessment will be applied to evaluate the certainty of evidence found within the proposed meta-analysis [51].

## Results

**Figure 1:**
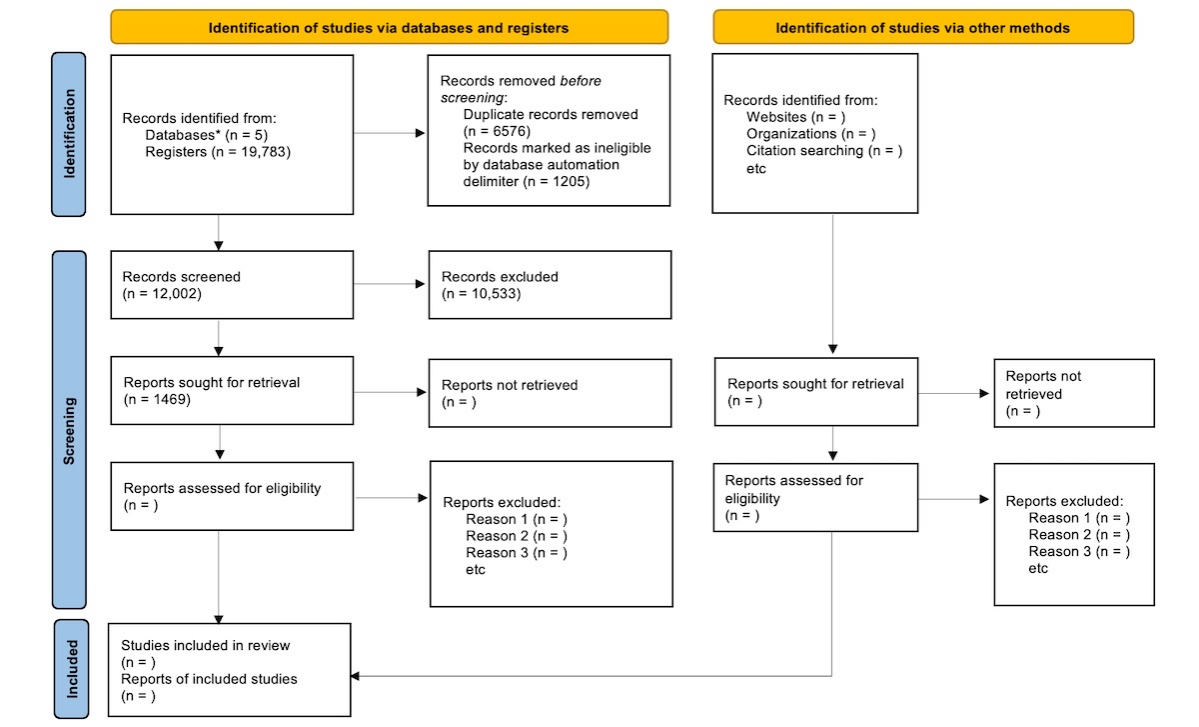
PRISMA (Preferred Reporting Items for Systematic Reviews and Meta-Analyses) flow diagram. *PsycINFO-OVID (n=5050); MEDLINE-OVID (n=3978); EMBASE-OVID (n=5631); CINAHL (n=5033); ProQuest Dissertation & Theses (n=91).

The literature search was completed on April 14, 2021. Following the removal of duplicates using the *synthesisr* R package, 12,002 articles were retained for initial title and abstract review. As of July 2021, title and abstract screening has been completed, with 1469 out of 12,002 (12.23%) studies included for full-text review. The PRISMA flow diagram is shown in Figure 1 [52]. Initial interrater reliability for the title and abstract review was 93.7% (761/12,002 conflicts, 6.34%). Full review is expected to be completed in the summer of 2021, with initial results expected for publication by early winter of 2021.

## Discussion

This paper describes the protocol for a meta-analysis and systematic review of the literature on the effectiveness of existing treatment approaches for military-related PTSD. The review will address gaps in the literature, including complexities of the clinical characteristics of PTSD, approaches of and diversities in implementing treatments, and population characteristics that may influence treatment outcomes. This comprehensive review aims to broadly substantiate evidence of PTSD treatment effectiveness to advance consensus guidelines for the treatment of military-related PTSD. The outcomes of this review will serve as a database of available evidence on the treatment of PTSD in military and veteran populations and begin to examine unanswered questions related to treating military-related PTSD.

## Abbreviations

DSM: Diagnostic and Statistical Manual of Mental Disorders
GRADE: Grading of Recommendations, Assessment, Development and Evaluations
PRISMA: Preferred Reporting Items for Systematic Reviews and Meta-Analyses
PTSD: posttraumatic stress disorder
